# Pharmacokinetic Interactions between Herbal Medicines and Drugs: Their Mechanisms and Clinical Relevance

**DOI:** 10.3390/life10070106

**Published:** 2020-07-04

**Authors:** Laura Rombolà, Damiana Scuteri, Straface Marilisa, Chizuko Watanabe, Luigi Antonio Morrone, Giacinto Bagetta, Maria Tiziana Corasaniti

**Affiliations:** 1 Preclinical and Translational Pharmacology, Department of Pharmacy, Health and Nutritional Sciences, Section of Preclinical and Translational Pharmacology, University of Calabria, 87036 Rende, Italy; laura.rombola@unical.it (L.R.); damiana.scuteri@unical.it (D.S.); strafacemarilisa@libero.it (S.M.); luigi.morrone@unical.it (L.A.M.); 2 Pharmacotechnology Documentation and Transfer Unit, Preclinical and Translational Pharmacology, Department of Pharmacy, Health and Nutritional Sciences, University of Calabria, 87036 Rende, Italy; 3 Department of Physiology and Anatomy, Tohoku Pharmaceutical University, 981-8558 Sendai, Japan; w-chizu@tohoku-mpu.ac.jp; 4 School of Hospital Pharmacy, University “Magna Graecia” of Catanzaro and Department of Health Sciences, University “Magna Graecia” of Catanzaro, 88100 Catanzaro, Italy; mtcorasa@unicz.it

**Keywords:** herb–drugs interactions, pharmacokinetics, herbal medicines, pharmacokinetic interactions

## Abstract

The therapeutic efficacy of a drug or its unexpected unwanted side effects may depend on the concurrent use of a medicinal plant. In particular, constituents in the medicinal plant extracts may influence drug bioavailability, metabolism and half-life, leading to drug toxicity or failure to obtain a therapeutic response. This narrative review focuses on clinical studies improving knowledge on the ability of selected herbal medicines to influence the pharmacokinetics of co-administered drugs. Moreover, in vitro studies are useful to anticipate potential herbal medicine-drug interactions. In particular, they help to elucidate the cellular target (metabolic or transporter protein) and the mechanism (induction or inhibition) by which a single constituent of the herbal medicine acts. The authors highlight the difficulties in predicting herbal–drug interactions from in vitro data where high concentrations of extracts or their constituents are used and pharmacokinetics are missed. Moreover, the difficulty to compare results from human studies where different kinds of herbal extracts are used is discussed. The herbal medicines discussed are among the best sellers and they are reported in the “Herbal Medicines for Human Use” section of the European Medicinal Agency (EMA).

## 1. Introduction

Therapeutic and adverse effects (ADRs) depend on a drug’s amount that reached systemic circulation and the site of action, and this is related to the dose, administration route and the drug’s pharmacokinetics (bioavailability, metabolism, distribution and clearance). Preclinical and clinical studies that were conducted to the approval rating of a new drug include data about the dose and administration route used to obtain the therapeutic effects without adverse reactions. Moreover, pharmacokinetic data collected in the above studies, along with the assessment of the long-term safety and efficacy of the drug (benefit–risk ratio), identify many factors, such as disease states, age, genetic polymorphisms and polypharmacy, that may modify pharmacokinetics of the drug and thus influence its responses. However, lack of therapeutic effect or unexpected unwanted side effects of a drug may also be due to the concomitant use of a medicinal plant. In particular, constituents in the medicinal plant extracts may influence drug pharmacokinetics, leading to drug toxicity or failure to obtain a therapeutic response depending on whether the drug reaches too high or inadequate tissue levels, respectively. Knowing if the components of the medicinal plant are able to induce or inhibit transporters or metabolic enzymes, it could be useful to predict potential pharmacokinetic interactions between herbal medicines and drugs. Thus, a number of in vitro studies have approached the potential of selected herbal extracts and/or specific constituents to induce or inhibit transporters or drug-metabolizing enzymes, mainly P-glycoprotein (P-pg) and cytochrome P450 (CYP450) isoforms. Furthermore, transporters belonging to the ATP-binding cassette transporters (ABC transporters) and solute carrier (SLC) transporters as well as phase I and II enzymes can also be targeted by plant constituents. Unfortunately, the translation of in vitro results in clinical data is arduous to achieve, then differences are often noted between the results of controlled clinical studies and in vitro results. Different factors can underlie this discrepancy. Mainly, the high concentrations of extracts or their constituents that are used in vitro to inhibit or to induce transporters or drug-metabolizing enzymes, are not obtained in humans after the administration of the conventional dose. Besides, some other factors to consider would be the modification of enzyme activity in the incubation setting induced by ionic strength, pH changes or by the solvent used to melt the extract. Furthermore, in vitro studies do not take into account the parameters, such as bioavailability, protein-binding properties or in vivo formation of metabolites. The lack of medicinal plant standardization makes the scenario more complex. In fact, in different studies, the same natural products may show quantitative and qualitative differences in the chemical composition. This review focuses on drug interaction with herbal medicines that are top selling [1] and several of them are reported in the “Herbal Medicines for Human Use” section of the European Medicinal Agency (EMA). The review is mainly concerned with herbal medicine–drug interactions at the level of metabolism, most of the literature data referring to medicinal plant interactions involving metabolic enzymes and carriers. Although the results obtained are not always mutually overlapping, it is not to be excluded that these natural extracts may induce drug pharmacokinetic interactions.

## 2. Results

### 2.1. Mechanisms of Action of Herbal Medicines-Drugs Interactions

The induction or inhibition of metabolic enzymes and transporters are the main mechanisms of action of the active compounds in the herbal medicines. It is well documented that a receptor-mediated mechanism is involved in the induction of drug-metabolizing enzymes and transporters, particularly orphan nuclear receptors, including pregnane X receptor (PXR) (Nuclear Receptor Subfamily 1 Group I Member 2, *NR1I2*) and constitutive androstane receptor (CAR) (Nuclear receptor subfamily 1 group I member 3 protein, *NR1I3)*. P-gp is expressed in normal human tissues such as liver, kidney, intestine and the endothelial cells of the blood–brain barrier. The apical (or luminal) expression of P-gp in these tissues results in reduced drug absorption from the gastrointestinal tract, enhanced drug elimination into bile and urine, and impeded the entry of certain drugs into the central nervous system. Likewise, the expression of CYP is ubiquitous. In the liver, the activation of PXR stimulates the expression of the CYP3A family members [2,3,4] and CYP2, including CYP2B6, CYP2C8, CYP2C9 and CYP2C19 [5]. Moreover, Phase II genes that are up-regulated by PXR ligands include members of the UDP-glucuronosyltransferase (UGTs), glutathione-S-transferase and sulfotransferase (SULTs) families. In the intestine, PXR stimulates the expression of ABCB1, while, in the liver, PXR stimulates the expression of organic anion transporting polypeptide (OATP)2B1 and multidrug resistance-associated protein (MRP)2 [5]. In the liver, CAR and PXR share many target genes, such as CYP3A, CYP2C, CYP28, UDP-glucuronosyl-transferases, glutathione S-transferases, and sulfotransferases, and drug transporters, including OATP1B1, MRP2 and ABCB1 [5]. Moreover, in different tissues PXR stimulates the expression of ABCB1, MRP2 and OATP1B1 [5,6]. Intriguingly, several studies indicate that the activation profiles of CAR and PXR is different among species [4,7]. Thus, for example, rifampicin activates human PXR but has virtually no activity on the rat or mouse receptors, while pregnenolone 16α-carbonitrile (PCN) activates mouse and rat PXR but it exhibits much less activity on human PXR [4]. Accordingly, the PXR activation profiles of these chemicals correlate closely with their CYP3A induction profiles in hepatocytes derived from these different species. These observations clearly suggest that attention should be paid in extrapolating data obtained in cultured rodent cells, or even in laboratory animals to humans, unless “humanized” transgenic mouse strains are used, expressing the human PXR or CAR instead of the endogenous receptor [8,9]. Additional NRs that regulate genes linked to drug absorption, distribution, metabolism and excretion are the bile acid-activated farnesoid X receptor (FXR, *NR1H4*); oxysterol activated liver X receptor (LXRα, *NR1H3*); fatty acid/eicosanoid-activated peroxisome proliferator activated receptor (PPARα, *NR1C1*), and retinoid-related orphan receptors (RORα, RORγ) (see [10]). Moreover, hepatocyte nuclear factor (HNF4α, *NR2A1*), a member of the NR superfamily, plays a synergizing role in the PXR- and CAR-mediated transactivation of drug pharmacokinetics and transporter-encoding genes (see [10]). Finally, the aryl hydrocarbon receptor (AhR) is a ligand-dependent transcription factor that can be activated by a structurally diverse range of synthetic chemicals but also through herbal extracts. The AhR is mainly involved in the induction of CYP1A isoforms (see [11]). Inhibition mainly concerns metabolic enzymes and can be generally divided into three categories: reversible, quasi-irreversible and irreversible inhibition (see [12]). The reversible inhibition is involved in drug interactions and occurs between the competition for CYP binding site between substrate drugs and inhibitors. Reversible inhibition can be further divided into competitive, non-competitive, uncompetitive, and mixed-type inhibition (see [12]). In particular, competitive or non-competitive inhibition is mainly involved in interactions between herbal medicines and drugs. Competitive inhibition happens between mutually exclusive substrates and inhibitors. A non-competitive inhibitor simultaneously binds different enzyme active sites. Mixed inhibition is a combination of competitive inhibition and non-competitive inhibition (see [12]). In contrast to the non-competitive inhibitors, uncompetitive inhibitors only bind the enzyme–substrate complex. The four types of enzyme inhibitions can be differentiated by monitoring Km and V_max_ values of the substrate.

#### 2.1.1. St John’s Wort (SJW)

Miliar stone of the herb–drug interactions refers to the capacity of St John’s wort (Hypericum perforatum; SJW) to induce the expression of several members of CYPs and P-gp (Table 1).

In particular, the National Institutes of Health (NIH) conducted the first pharmacokinetic study that was suggested for SJW acting as an inducer of CYP3A4. That study was performed to verify whether hypericum extract affects the plasma level of the HIV protease inhibitor indinavir, a substrate of CYP3A4. The results of that study indicate that a 2-week treatment with SJW reduced the area under the curve (AUC) of indinavir by a mean of 57% and decreased the extrapolated 8-h indinavir through by 81% in healthy volunteers [13].

In the same period, in two heart transplant patients, Ruschitzka et al. [14] measured a decrease in the plasma concentration of cyclosporine and acute rejection after the administration of hypericum extract. The causative role of SJW in decreased levels of the drug was suggested by the finding that cyclosporine concentrations returned within the therapeutic range when SJW treatment was discontinued with no further episodes of rejection [14]. A Public Health Advisor was immediately issued by the Food and Drug Administration (FDA) based on the data of Piscitelli et al. [13] on the risk of interaction between indinavir, or other drugs metabolized by cytochrome P450, and hypericum extract [13]. Indeed, previous to Piscitelli’s results, it was shown that SJW affects the pharmacokinetics of P-gp substrates, but, initially, this study was underestimated [15] (Table 1).

In particular, Johne and colleagues [15] observed no effects after the acute administration of the extract on the pharmacokinetics of digoxin but they measured a statistically significant decrease in digoxin AUC_(0-24)_ by 25%, and maximum concentration (C_max_) of 26% after 10 days of treatment with SJW, whereas no effect was observed for time for maximal concentration (T_max_) and the terminal half-life of elimination (t_½_) [15]. Oral bioavailability and the renal clearance of digoxin are regulated by P-gp activity. Thus, it was speculated that SJW also induced this protein. Thence, several clinical studies have been published supporting this initial evidence that has suggested that SJW acts as an inducer of CYP metabolic and P-gp pathway. Quickly, experimental in vitro and in vivo studies elucidated the molecular mechanism underling the ability of SJW to decrease oral availability and/or accelerate the metabolism of drugs co-administered with the extract. The Glaxo Wellcome Research and Development group identified orphan nuclear receptor PXR as the target of SJW, the same as rifampicin, a well-known activator of this receptor and CYP3A4 expression. Hyperforin, one of the major constituents present in the dried flowering tops or aerial parts of SJW [16], mediated transactivation and coactivator recruitment by steroid and xenobiotic receptor (SRX) [17] and was an activator of PXR with a half-maximal effective concentration (EC_50_) of 23 nM [18], making it one of the most potent PXR activators to be reported to date. This value is lower than of 200 nM. This concentration is the value of the steady-state plasma levels of hyperforin that is measured in humans after administration of the standard dose of SJW (3 × 300 mg, daily) and is used to treat depression [19]. Successively, it was demonstrated that genes other than CYP3A4 [2,3,4] are induced by PXR in humans following activation by xenobiotics, including CYP2B6 [20], CYPlA1 and 1A2 [21], CYP2C8 and 2C9 [22] as well as ABCB1 [23] and MRP2 [24]. Indeed, these in vitro data were overlapped with original evidence that the administration of SJW extract at a dose of 300 mg three times per day for 14 days to eight healthy male volunteers enhances the expression of CYP3A4 (1.5-fold) and duodenal P-gp (l.4-fold). These results were obtained by western blot analysis of duodenal biopsy specimens taken before and after treatment, and it was shown that it increases the functional activity of hepatic CYP3A4 (1.4-fold), as evaluated by the 14C-erythromicin breath test [25]. Moreover, in peripheral blood lymphocytes of healthy volunteers, SJW increased expression and enhanced the drug efflux function of P-gp [26]. Another in vivo study supports the evidence that hyperforin is the active constituent responsible for the inductive effect of SJW in humans. In fact, administration of a conventional SJW extract (900 mg/day for 2 weeks), under a stable cyclosporine regimen to renal transplant patients, resulted in a significant decrease in cyclosporine AUC values and required a substantial increase in cyclosporine dose to maintain sufficient immuno-suppression [27]. Instead, treatment with SJW free of hyperforin by supercritical carbon dioxide extraction did not significantly affect cyclosporine pharmacokinetics and no dosage adjustment was necessary [27]. Successively, it was demonstrated that the induction of CYP3A and P-gp by SJW, in healthy volunteers, depends on hyperforin concentration in the different type of extracts used [28,29,30], and, in particular, it was suggested that a daily dose of 1 mg of hyperforin is a critical cut-off for clinically significant interactions [31]. A recent randomized, double-blind, parallel-arm, clinical trial enrolling 16 healthy volunteers has evaluated the effect of SJW on fentanyl, the latter being metabolized by hepatic CYP3A4 and transported by P-gp [32]. Under these circumstances, the pharmacokinetics and pharmacodynamics of fentanyl do not result affected. Moreover, it has been demonstrated that the duration of treatment influences the potential of interaction by SJW. In fact, some data show that a treatment period of 14-days with SJW [33,34] is needed to produce significant effect on CYP3A4 activity while a short-term one doesn’t induce any effect [33,35].

Therefore, several controlled clinical studies have been issued proving that SJW significantly modifies the pharmacokinetics of drugs that are substrate of CYP3A, 2C9, 2C19, 2E1 or transported by P-gp or both, including midazolam [33], simvastatin [36], amitriptyline [37], chlorzoxazone [38,39], methadone [40], ethinyl estradiol/norethindrone combination oral contraceptives [41,42], fexofenadine [43], warfarin [44], imatinib [45], omeprazole [46], mephenitoin [47], tacrolimus [48], verapamil [49], voriconazole [50], talinolol [51], gliclazide [52], nifedipine [53], ketamine [54], zolpidem [55], bosentan [56], ambrisentan [57] and docetaxel [58] (Table 1).

Intriguingly, SJW interaction was expanded to multidrug resistance ATP-binding cassette (ABC) proteins and solute carrier (SLC) transporters as reported initially in a case report [59]. The authors indicated an increase in low-density lipoprotein (LDL) cholesterol and total cholesterol levels in a hypercholesterolemic patient stabilized with rosuvastatin. The patient had taken an herbal supplement, including SJW plus spirulina and rosemary, 5 months before the drug. The serum lipid levels lowered after the discontinuation of the herbal supplement, indicating that the latter plays a causative role for the reduced efficacy of the statin [59]. It is tricky to elucidate the molecular mechanisms underlying this interaction by this kind of herbal supplementation (SJW associated with rosemary and spiruline) and the number of transporters involved indicating rosuvastatin disposition. Moreover, 80% of an orally administered dose of rosuvastatin in humans is recovered unchanged in the feces; therefore, the metabolic transformation (mainly by CYP2C9) plays a minor role in rosuvastatin clearance. BCRP plays an important role in limiting the intestinal absorption of rosuvastatin [60,61]; indeed, rosuvastatin is a substrate of bile canalicular (MRP2, P-gp, BCRP) sinusoidal uptake (OATPIB1, OATP1B3, and OATP2B1) transporters [60,61]. These preliminary data did not permit to clearly establish a role for SJW in the reported interaction with rosuvastatin. More recently, hypericum extract (twice daily for 21 days) has been shown to induce a slight reduction of metformin renal clearance, no effect on steady-state concentration of the drug but a statistically significant increase in glucose tolerance in human volunteers (ClinicalTrials.gov:NCT01726764) [62]. Metformin is eliminated unchanged in the urine [63] and its distribution is regulated by multidrug and toxin extrusion (MATE) (SLC47A1-2), plasma membrane monoamine transporter (PMAT) (SLC29A4) and organic cation transporter (OCT) (SLC22A1-3) transporters [64], particularly the isoforms OCT1 and OCT3 mediate uptake in the liver [65]. The lack of effect by hypericum extract on metformin pharmacokinetics and no differences in OCT1 mRNA concentration, measured in peripheral blood cells of the subjects, leave one to hypothesize no influence of SJW on SLC transporters. A lack of pharmacokinetic interaction was also observed in healthy volunteers after the co-administration of hypericum extract (three times daily for 14 days) with repaglinide [66], a hypoglycemic anionic drug that is taken up in the liver by OATP1B1 transporter [67]. Intriguingly, it was observed that the other PXR agonist rifampicin modifies the AUC of metformin [68] and repaglinide and increases OCT1 mRNA levels [69]. The later data indicate that the scenario looks more complicated than was initially suspected. Moreover, although it is worldwide known that SJW is able to induce metabolic enzymes and P-gp, there was a case report in 2012 [69] where the plasma concentration of clozapine, in a schizophrenic patient, suddenly decreased and worsened her clinical condition because hypericum extract was taken as self-medication [70]. As indicated in an EMA report of risk assessment [EMA/HMPC (2018). Assessment report on Hypericum perforatum L., herba Draft EMA/HMPC/244315/2016] and clearly updated in the review of Nicolussi and colleagues [6], the use of SJW products is recommended at a safety threshold of maximum 1 mg of hyperforin per day [6]. Special care should be taken in the use of high-hyperforin SJW medicinal plant and among botanicals and food/dietary supplements [6].

Although the hyperforin of SJW was the first herbal constituent reported to activate PXR, other herbal medicines, including Ginkgo biloba and garlic extracts, have been reported to activate this nuclear receptor and induce in vitro transporter proteins and/or metabolizing enzymes (Table 2 and Table 3).

Nevertheless, often, the clinical relevance of these data is questionable due to the lack of correlation between the high concentrations of extracts or their constituents necessary to activate nuclear receptors and to induce transporters or drug metabolizing enzymes in vitro, with those effectively obtained at standard dosage in humans. It is important to consider that in vitro studies do not include important factors such as bioavailability or the generation of active metabolites. All these limitations of in vitro studies may clarify the gap, often documented, between outcomes predicted by in vitro studies and the data of controlled clinical studies. Moreover, contrasting results are often included in clinical studies, thus making the forecasting of the clinical relevance even more confused (Table 2 and Table 3). These differences arise from the study design (dosage, duration of treatment), the variability in phytochemical composition of commercially available herbal supplements, the selection of substrate drug used to foretell enzyme activity as well as the choice of pharmacokinetics performed to assess the occurrence of herb–drug interactions (e.g., single-time point versus canonical AUC analysis). Lastly, often, the constituent/s responsible for the reported activation of nuclear receptors are not identified and only a hypothesis about the candidate constituent/s can be discussed.

#### 2.1.2. Ginkgo Biloba

Initially, it has been highlighted in an in vitro cell-based luciferase reporter gene assay that an extract of Ginkgo biloba activates human PXR, in a concentration-dependent manner [71]. This extract included concentrations of ginkgolide A, B, C, and J, bilobalide and flavonols similar to those found in the standardized extract EGb 761 [71]. In particular, G. biloba extract at 200 µg/mL increased by 9.5-fold human PXR activation [71] and the expression of PXR target genes, i.e., CYP3A4, CYP3A5 and ABCB1 (encoding P-gp) in human LS180 colon adenocarcinoma cells [71]. Successively, Li and colleagues [71] confirmed that G. biloba extract (EGb 761, 100 µg/mL) activates human PXR by identifying the constituent/s responsible for this effect [72]. Accordingly, in human HepG2 cells, cell-based reporter assays indicated that ginkgolide A (GA; 50 µM) and ginkgolide B (GB; 50 µM) activate PXR; quercetin (25 µg/mL) and kaempferol (20 µg/mL) activate PXR, CAR, and aryl hydrocarbon receptor (AhR), whereas bilobalide (BB; 50 µM) does not induce effects on these nuclear receptors [72]. In particular, in human primary hepatocytes, it was shown that only EGb 761, GA, and GB were able to induce CYP3A4, CYP2B6, UGTI A1, ABCB1 and MRP2, whereas BB and the flavonoids kaempferol and quercetin were inactive [72]. More recently, molecular docking analysis has showed that the PXR binding energy was great for GA and GB [73]. The authors compared the GA effect with hyperforin, pointing out that the ginkgolide was less powerful (EC_50_ = 16.2 µM) than hyperforin (EC_50_ = 0.02–0.2 µM) but both showed similar efficacy (Emax = 11.5 fold for GA vs. 6- to 12-fold for hyperforin) [73]. Specifically, it was documented that the flavonoidic fraction of EGb761 inhibits the enzyme activity of CYP 3A4, 2C9, 1A2, 2El with Ki between 4.9 and 55 microg/mL [74]. Not exhaustive and contrasting data were obtained in vivo after the treatment of rats with G. biloba, where it was shown that the extract increases metabolic [75] and inhibits P-gp activity [76]. The administration of G. biloba extract (100 mg/kg for 5 days) significantly reduced the AUC (0-12) of tolbutamide by 53% in low-protein diet-fed rats and by 38% in normal rats [75]. Conversely, treatment with G. biloba extract for 30 days with a dose of 100 mg/kg increased tissue venlafaxine levels in kidney tissue measured as a tissue/serum drug ratio, while a dose of 200 mg/kg of extract increases fluoxetine availability in liver, kidney and brain tissues [76]. Consequently, the extrapolation of the in vitro or rodent results to human situation is complicated and, in fact, contrasting evidence of interactions comes also from clinical studies using G. biloba. Treatment with G. biloba extract (240 mg/day for 2 weeks), in healthy volunteers, significantly decreased by 17% the AUC of alprazolam, CYP3A4 substrate, but did not affect the t_½_ [77]. Accordingly, similar results were shown in another study in healthy human volunteers treated with G. biloba extract (240 mg/day for 4 weeks) where a decrease in C_max_ by 31% and in the AUC by 34% for midazolam, CYP3A4 substrate was observed [78]. Conversely, G. biloba extract (240 mg/day for 4 weeks) did not modify the serum 1-hydroxymidazolam midazolam ratio (used as an in vivo index of CYP3A-mediated drug metabolism) either in young [38] or in elderly [39] healthy subjects. However, successively, it has been shown that the use of a single post-dose midazolam concentration is of limited usefulness to predict CYP3A4-mediated drug interactions [79]. Further data were obtained by Uchida et al. [79] in male healthy volunteers where a significant decrease in the oral clearance (26%) and an increase in the AUC (25%) for midazolam were observed after treatment with G. biloba extract (EGb 761; 360 mg/day for 4 weeks) [80]. It can be presumed that the dose (360 vs. 240 mg) and the type of ginkgo extract utilized were responsible of the contrasting results observed with midazolam. Interestingly, concomitant oral administration of nifedipine (CYP3A substrate) and G. biloba extract did not modify the plasma concentration of the drug except in two of eight human subjects [81]. In these volunteers, G. biloba extract (240 mg) induced a doubled nifedipine C_max_ and the appearance of long-lasting and intense side effects, such as dizziness, hot flashes and headaches [81], suggesting that ginkgo may act as an enzyme inhibitor during acute treatment. All these clinical results show that an interaction between G. biloba extract and CYP3A substrate drugs cannot be anticipated, but never excluded; however, it is difficult to conclude if an induction or inhibition of drug metabolizing enzymes or transporters occurs. Greenblatt and colleagues [81] observed that the co-administration of a single dose (360 mg) of G. biloba extract did not alter the pharmacokinetics of flurbiprofen, CYP2C9 substrate [82]; likewise, treatment for 7 days with G. biloba extract did not affect the pharmacokinetics of warfarin, another CYP2C9 substrate drug [83]. Conversely, tolbutamide AUC significantly decreased after treatment with EGbl 761 extract (360 mg/day) for 4 weeks [80]. Overall, these data would suggest that the duration of treatment may be responsible for the differences observed in healthy volunteers.

Contrasting results were also obtained on the effects of ginkgo in healthy volunteers who were taking CYP2C19 substrate drugs. The induction of CYP2C19 activity observed by Hellum et al. [84] in human cultured hepatocytes could explain the effects observed by Yin et al. [83] in healthy Chinese subjects where the treatment for 12 days with G. biloba extract (280 mg/day) produced omeprazole hydroxylation in a CYP2C19 genotype-dependent manner [85]. Moreover, in either CYP2C19 extensive or poor metabolizer, G. biloba extract (240 mg/day for 12 days) did not induce effects on pharmacokinetics of voriconazol, a substrate of CYP2C19 [86]. Similar negative results were shown by Zuo and co-workers [85] on the CYP2C19-mediated N-demethylation of diazepam in healthy volunteers; based on the finding that 90% confidence intervals of the mean ratios of AUC_(0-408)_ and C_max_ of diazepam, both in the absence and presence of G. biloba extract, were within the no-effect boundary of 80–125% (equivalence range) [85]. The authors concluded that no clinical robust interaction occurs with the extract [87]. Of interest, the analysis of tabulated arithmetic means and standard deviations of AUC_(0-408)_ and C_max_ indicates that G. biloba extract supplementation leads to a statistically significant decrease by 37% and 25% of diazepam AUC and C_max_, respectively, and to a significant 17% increase in N-demethyldiazepam AUC_(0-408)_ [87]. Of course, the statistical analysis performed by Zuo and co-workers [85] is appropriate as the confidence intervals convey a probability of the magnitude of the interaction [87]. However, the application of tests gives information on whether or not the systemic exposure differences are statistically significant (*p* < 0.05) but not whether they are clinically pertinent. Nevertheless, in the studies with medicinal plants, a number of factors, including the duration of treatment, the variability of the extract composition and the limited number of subjects recorded (Table 2), could influence any statistically significant changes in the pharmacokinetic parameters. In fact, it cannot be excluded that a reduction in the AUC by about 35% may significantly influence the therapeutic response of a drug; this may not be the case with diazepam as its metabolites remain active. In vitro results showing the induction of G. biloba on CYP2B6 [72], were validated by a clinical case that showed concomitant virologic failure with a marked decrease in the plasma concentrations of efavirenz, a CYP2B6 substrate drug, in an HIV-infected patient who also took G. biloba extract for some months [88]. The last remark would indicate that the intake of G. biloba decreases oral bioavailability and/or improves the systemic hepatic clearance of efavirenz, thus suggesting a serious precautionary note on ginkgo supplementation by patients who also take drugs substrate of CYP2B6. Moreover, no information on the dosage and the type of extract used by the patient described by Wiegman and colleagues [86] restricts the power of this observation and does not allow any generalization to be made [88]. Actually, treatment with G. biloba (120 mg twice daily for 14 days) in healthy volunteers did not significantly change the pharmacokinetics of bupropion, CYP2B6 substrate drug, although the extract significantly increased C_max_ and reduced t_½_ of hydroxybupropion [89].

Reduced plasma levels of efavirenz reported by Wiegman et al. [86] may result from an induction of CYP3A4 also implicated in the drug metabolism [88]. Although G. biloba extract increased the expression of MDR transporter in vitro [71,72], treatment with the extract did not alter the pharmacokinetics of two substrate drugs for P-gp, digoxin [90] and fexofenadine [78]. Surprisingly, in healthy volunteers, the administration of G. biloba extract (360 mg/day for two weeks) significantly increases the C_max_ and AUC of another substrate drug for P-gp, i.e., talinolol, but it does not affect its t_½_ [91], suggesting that G. biloba operates as an inhibitor of intestinal P-gp. Incidentally, by in vitro results [92], it can be supposed that flavonoids in the extract may have inhibited intestinal efflux transport of talinolol, thus effectively increasing its oral bioavailability. The involvement of P-gp transporter in G. biloba–drug interaction was successively observed by Blonk and colleagues [93] with raltegravir, a weak P-gp substrate [94]. In healthy volunteers, G. biloba extract (120 mg twice daily for 14 days) induced a geometric mean ratios (90% confidence intervals) difference (1.44 vs. 1.21) of C_max_ and AUC_(0–∞)_ when raltegravir was administered alone or with the extract trials [93].

Intriguingly, Zhou and colleagues [93] have demonstrated that the concentrations of ginkgolides necessary to inhibit or induce CYP isoforms in vitro were well above the concentrations obtained after intake of standard dose of the extract [95]. Incidentally, in human liver microsomes, hydrolysed form of GA, GB and GK tested on the activity of CYP1A2, 2B6, 2C8, 2C9, 2C19, 2D6 and 2E1 showed a value of IC_50_ of 10 µg/mL, too high to be reached in humans [95]. Indeed, in cryopreserved human hepatocytes, the mRNA expression and enzyme activities of CYP1A2, 2B6 and 3A4 were statistically increased only after incubation for 48 h with this high concentration (10 µg/mL) of the ginkgolides [95]. Consequently, in healthy volunteers, the administration of EGb 761^®^ at standard dosage (120 mg twice daily or 240 mg once daily, for 8 days) did not affect the plasma concentration of caffeine, tolbutamide, omeprazole, dextromethorphan and midazolam used as a substrate of CYP1A2, CYP2C9, CYP2C19, CYP2D6 and CYP3A, respectively [96]. Similar results were achieved successively; in fact, no changes in the pharmacokinetics of cilostazol, CYP3A4 and CYP2C19 substrate drug, or its metabolites 3,4-dehydrocilostazol and 4′-trans-hydroxycilostazol, were observed after treatment with G. biloba extract (Ginexin^®^) (80 mg 120 mg twice daily for 7 days) in healthy volunteers [97]. However, a larger dose of G. biloba extract (360 mg for 14 days) in healthy volunteers weakly reduced AUC(0–∞) (10%) and C_max_ (29%) and increased the distribution (volume of distribution (Vd) 31.95%) and elimina½ion (clearance (CL), 6.48%) of atorvastatin [98]. Atorvastatin is mainly transformed by CYP3A4 [99] but G. biloba extract treatment does not modify the atorvastatin metabolic pathway [98]. Different results were obtained by Dai and colleagues [98] (Chinese Clinical Trial Registry:ChiCTR-TTRCC-12002801), who observed an increase in the AUC_(0–∞)_ (36%) and C_max_ (32%) of simvastatin after treatment with G. biloba extract (120 mg twice daily for 14 days) [100]. Statins are taken up into hepatic cells mainly by OATP1B1 transporters and it is known that genetic polymorphism of this transporter is strongly associated with statins uptake; in particular, the 521CC genotype has been shown to elevate plasma concentrations of different statins [101,102]. It could be supposed that the decrease in the bioavailability of statins is associated with the capacity of G. biloba extract to also induce OATP1B1 activity if no effects have been observed on the cholesterol-lowering efficacy of these drugs [98,100]. Moreover, although, in the study of Guo and co-workers [96], subjects with the 521TT genotype are included, the data should be achieved on the activity or expression of intestinal and hepatic transporters after treatment with G. biloba in humans [98]. In conclusion, the in vitro results obtained by Zhou and colleagues [93] indicated that ginkgolides are active on metabolic enzymes only at a high concentration [95], not achievable after the administration of standard doses of G. biloba extract in a healthy subject, and these results can explain the lack of effect of the extract on pharmacokinetics of co-administered drugs (Table 2). However, more recent studies highlight the capacity of gingkolides to affect intestinal and/or hepatic transporters suggesting some precaution in G. biloba extract administration with their substrates.

#### 2.1.3. Allium Sativum

Conflicting data are often obtained in controlled clinical studies for garlic (*Allium sativum*) interactions. These inconclusive results are likely due to differences in the duration of treatment and/or the use of different garlic-derived materials (Table 3). In fact, many types of commercial garlic products are indeed available, including aged garlic extract (AGE), garlic essential oil and garlic powder. AGE includes water soluble sulphur compounds such as S-allylmercaptocysteine (SAMC) and S-allylcysteine (SAC) and small amounts of oil-soluble allyl sulphides. Garlic essential oil includes only oil-soluble sulfur components, such as diallyl trisulfide (DATS) and diallyl sulfide (DAS), but no allicin or water-soluble fraction. Garlic powder contains alliin and a small amount of oil-soluble sulphur compounds. The amounts of alliin present in different strains of garlic ranging from 2.8 to 7.7 mg·g^−1^ [103]. Garlic and garlic powder do not contain allicin but it is produced through an enzymatic reaction catalysed by alliinase. This enzyme is present in large amounts in garlic cloves: at least 10% of the total protein content (10 mg·g^−1^ fresh weight). Specifically, alliinase is localized in vascular bundle sheath cells, whereas alliin is compartmentalized in mesophyll cells. Wetting the powder or crushing the clove induces tissue disruption and consequently alliin is released from compartments and interacts with alliinase leading to the synthesis of allicin in few seconds. Garlic cloves yield about 2.5 to 4.5 mg of allicin per gram of fresh weight when crushed [103]. Several in vitro studies refer to the potential of garlic, or selected garlic constituents, to inhibit P450 enzymes but they do not allow us to elucidate data achieved in controlled clinical studies using garlic. It was previously reported, by using an in vitro assay, that both fresh garlic extracts and various commercial garlic products inhibited the activities of cytochrome P4503A4, 2C9 *1, 2C19, 3A5, and 3A7 but not that of CYP2D6 and exhibit a modest effect on P-gp activity [104]. Selected water-soluble constituents of aged garlic were analysed for their ability to inhibit the activity of CYP2C9, 1A2, 2CJ1, 2B6, 2D6, and 3A in human liver microsomes; of these, only S-allyl-L-cysteine and S-methyl-L-cysteine at 100 µmol/L produced a modest inhibition of CYP3A, reducing activity to 20–40% of the control [105]. Considering the limitations relative to in vitro studies, these data would indicate that garlic may inhibit during acute dosing. However, more helpful information about the in vivo effects of garlic on drug metabolizing enzymes may be inferred from animal studies that elucidated the molecular mechanisms underlying the capacity of garlic or related compounds, including diallyl sulfide (DAS), diallyl disulfide (DADS) and its CYP2El-derived metabolites, diallyl sulfone (DASO2) and diallyl sulfoxide (DASO), to reduce the incidence of a number of chemically induced tumors in animal models. However, in addition to efficiently inhibiting CYP2El in rat liver, DADS and DAS also had the capacity to induce CYP enzymes, including CYP3A, CYP1A and CYP2B families, and phase II detoxification enzymes such as gluthatione S-transferase (GST), NAD(P)H:quinone oxidoreductase (NQO), epoxide hydrolase (EH) and UDP-glucuronosyltransferase (UGT) in rat liver [106,107,108]. More recently, Berginc and colleagues [106] observed in vitro that AGE induces an increase in the activities of secretory (P-gp) and absorptive (OACT) transporters [109]. Incidentally, Asdaq and Inamdar [107] have shown that oral administration of garlic homogenate (250 mg/kg for 4 week) increases pharmacokinetics of propranolol in rats [110]. Moreover, in rodents, oral administration of DATS, 10 or 20 mg/kg/day for 15 days, modified the structure of the lower small intestine and decreased the C_max_ and AUC_0–24_ of dipyridamole [111]. The latter crosses biological membranes as a lipophilic drug and has no effect on the accumulation of rhodamine 123 and daunorubicin, typical fluorescent P-gp substrates [112]. From this evidence, it is possible to speculate that DATS reduced the bioavailability of dipyridamole by the modification of the dissolution rate and intestinal absorption of the drug. Pre-treatment or co-administration with DATS does not modify the intravenous pharmacokinetics of dipyridamole [111], suggesting that no induction of metabolic enzymes is involved. These in vitro and in vivo studies have been using doses (and concentrations) of garlic higher than those usually employed by humans; however, it is possible to speculate that garlic induces drug-metabolizing enzymes or modify intestinal absorption of drugs after chronic administration in humans. Actually, only few controlled clinical studies have been performed to assess the potential of garlic supplementation on drug pharmacokinetic parameters. Two studies have used a single time-point and phenotypic metabolic ratios to calculate whether the long-term (28 days) supplementation of garlic oil affected CYP2D6, CYP1A2, CYP2E1, or CYP3A4 activity in young and elderly subjects. Probe drug cocktails of midazolam, chlorzoxazone, debrisoquine and caffeine were administered before and at the end of supplementation. Pre- and post-supplementation phenotypic ratios were determined for CYP1A2, CYP3A4, CYP2E1, and CY2D6 using paraxanthine/caffeine serum ratios (6-h), 1-hydroxymidazolam/midazolam serum ratios (1-h), 6-hydroxychlorzoxazone/chlorzoxazone serum ratios (2-h), and debrisoquine urinary recovery ratios (8-h), respectively. Comparisons of pre- and post-phenotypic ratios indicated that garlic oil inhibited CYP2El activity by approximately 39% in young [37] and 22% in elderly [38] subjects. No effects were observed on CYP1A2, CYP3A4 or CY2D6 after garlic oil supplement either in young and elderly subjects [37,38], at least as calculated by single-time point analysis. Similar results were also achieved by Markowitz and co-workers [110], who failed to demonstrate the modification of the pharmacokinetics of CYP2D6 and 3A4 substrate drugs by using garlic powder tablets (kwai) [113]. In fact, garlic intake (3 × 600 mg tablets twice daily for 2 week) did not modify the AUC, C_max_, T_max_, t_½_, of alprazolam, CYP34 substrate drug, nor the urinary dextromethorphan/dextrorphan ratios used as an index of CYP2D6 activity [113]. This evidence would indicate that garlic supplements have a low risk for CYP-mediated herb-drug interactions; however, quite different results have been previously shown by Piscitelli and co-workers [114]. Specifically, in a clinical study, they evaluated the effect on the pharmacokinetics of saquinavir after treatment for 21 days with garlic powder caplets, administered twice daily. It was highlighted that garlic supplementation decreased drug C_max_ by 54%, AUC by 51%, and levels at 8 h after dosing (post dose C_8_) by 49% [114]. Moreover, saquinavir pharmacokinetics did not return to baseline values after a 10-day washout period; indeed, the mean AUC returned to 65%, the C_max_ to 61% and the C_8_ to 70% of baseline values, respectively. Because the AUC for saquinavir did not return to baseline values after the washout period, a direct impairment of absorption in the gastrointestinal tract can be supposed. More recently, Berginc and colleagues [115], summarising in vitro and in vivo data, tried to explain the effect of garlic on HIV-protease inhibitor pharmacokinetic. These authors suggested that garlic phytochemicals bind different sites on P-gp respect to saquinavir and thus natural compounds enable allosteric modulation resulting in an increase in the saquinavir efflux responsible for the decrease in drug bioavailability observed by Piscitelli et al. [115]. Furthermore, by comparison of saquinavir and darunavir effects on P-gp binding sites, the same authors pointed out that darunavir and phytochemicals in AGE bind the same sites, suggesting that no pharmacokinetic modification is expected with darunavir [115]. The divergent results achieved by Piscitelli et al. [114] and Markowitz et al. [113] (Table 3) could be attributed to a different dosing period (3 weeks versus 2 weeks) used. This hypothesis appears to be supported by another study, indicating that the intake of garlic capsules over 4 days did not modify significantly the single-dose pharmacokinetics of ritonavir (a drug with a high binding affinity for P-gp and CYP3A substrate) in healthy volunteers, although there was a trend for a decrease in ritonavir concentrations [116]. Compellingly, the authors had also indicated that two HIV-infected patients taking garlic or garlic supplements for more than 2 weeks showed serious gastrointestinal toxicity after beginning ritonavir-containing antiretroviral therapy (400 or 600 mg twice daily); the symptoms disappeared after discontinuing garlic or ritonavir [116]. However, the lack of data regarding plasma levels of ritonavir when administered with garlic constituents does not allow for speculation about the possible underlying mechanism. Successively, it has been indicated that garlic supplementation (600 mg tablets twice daily) does not significantly alter the disposition of docetaxel, a CYP3A4 substrate drug [117]. However, in patients carrying a CYP3A5*1A allele, on average, over a 12-day period, garlic decreased the clearance of docetaxel [117]. These data suggest that the genotyping of drug-metabolizing enzymes that exhibit clinically relevant polymorphisms should represent an integral part of herb–drug interaction studies to overcome some inconsistent results obtained from clinical studies. Overall, what can be anticipated is that special attention must be given when garlic supplements are employed by patients concurrently treated with drugs whose disposition depends on P-gp and/or CYP3A and CYP2E1 (Table 3).

### 2.2. Clinical Evidence of the Herbal–Drug Interaction Mediated by the Inhibition of Drug-Metabolizing Enzymes and/or Transporters

#### 2.2.1. Hydrastis Canadensis

Many studies have documented the potential of goldenseal (*Hydrastis canadensis*) extracts to inhibit the activity of cytochrome P450 enzymes leading to the concept that goldenseal extracts inhibit, at least in vitro, several isoforms of cytochrome P450 involved in drug disposition, for example, 3A4, 2E1, 2C8 and 2D6 [118,119,120,121]. Extracts of goldenseal, containing approximately equal concentrations (about 17 mM) of two methylenedioxyphenyl alkaloids hydrastine and berberine, in human hepatic microsomes, inhibited CYP2D6-mediated bufuralol 1′-hydroxylation, CYP2C9-mediated diclofenac 4′-hydroxylation and CYP3A4-mediated testosterone 6ß-hydroxylation activities with IC_50_ values of 0.66%, 0.98%, and 0.18%, respectively [118]. In particular, the inhibition of testosterone 6ß-hydroxylation activity by either goldenseal or hydrastine is non-competitive and inactivation of CYP3A4 probably arises from the methylenedioxyphenyl moiety of hydrastine interacting with the heme iron of the enzyme to generate a stable heme adduct [118]. In turn, in human liver microsomes, the inhibition of CYP2E1and CYP2C8 activities by goldenseal extracts was also indicated [119,120]. In particular, the alkaloids hydrastine, berberine and canadine seem to be implicated in the strong inhibition of CYP2E1 by goldenseal. These compounds inhibited CYP2E1 with Ki values ranging from18 µM for berberine to 2.8 µM for hydrastine [120]. Moreover, methanolic and aqueous extracts of goldenseal inhibited CYP2D6 activity in human liver microsomes with IC_50_ of 6.3 and 6.7 µg/mL, respectively [121]. The capacity of goldenseal to exhibit in vitro inhibitory activity on CYP3A4, drove some studies to examine in humans the influence of goldenseal administration on the disposition of CYP3A4 substrate drugs. Initially, Sandhu and colleagues [122] evaluated the effects of goldenseal (1140 mg, twice daily for 14 days) on the pharmacokinetics of indinavir, administered per os at the single dose of 800 mg. No significant changes in C_max_, T_max_, oral clearance, and the t_½_ of indinavir were observed, suggesting that interactions between drugs metabolized by CYP3A4 and goldenseal are unlikely [122]. However, after the oral intake of clinical doses, indinavir induces a dose-dependent kinetics probably due to saturable first-pass and systemic clearance [123]. Consequently, it is possible to speculate that, after an 800-mg oral dose, the saturation of indinavir metabolism might have reduced a further increase in oral and/or hepatic availability by goldenseal.

The first proof that goldenseal inhibits drug metabolism in vivo is derived from a study in which single-time point phenotypic metabolic ratios were used to determine whether the long-term supplementation of goldenseal (900 mg, three times daily for 28 days) affected CYP3A4/5, CYP2D6, CYP1A2, or CYP2E1 activity in healthy volunteers [124]. In that study, pre-supplementation and post-supplementation phenotypic trait measurements were measured for CYP2D6, CYP1A2, CYP2E1 and CYP3A4/5 through the use of debrisoquin urinary recovery ratios (8-h collection), paraxanthine/caffeine serum ratios (6-h sample), 6-hydroxychlorzoxazone/chlorzoxazone serum ratios (2-h sample) and-hydroxymidazolam/midazolam serum ratios (1-h sample), respectively. Comparisons of pre-supplementation and post-supplementation phenotypic ratio means showed that goldenseal produces a significant inhibition (about 40%) of CYP3A4/5 and CYP2D6 activity but not of CYP2E1 or CYP1A2 activity [124]. Follow-up investigation of the same authors, by use of conventional concentration-time profiles and AUC values to evaluate the effects of goldenseal on the pharmacokinetics of midazolam (used as a CYP3A sensitive probe), supported these early results. In fact, it was noted that supplementation with goldenseal (1.323 mg, three times daily) for 14 days significantly increased the C_max_, (by 41%), the AUC_(0-∞)_ (by 62%) and the t_½_ (by 57%) of orally administered midazolam with a concomitantly significant reduction of apparent oral clearance (by 36%) [125]. These data indicate that goldenseal may increase the oral availability and/or reduce the systemic hepatic clearance of CYP3A substrate drugs creating a severe risk of adverse drug effects and toxicity in patients treated with CYP3A4 substrate drugs showing narrow therapeutic indices (Table 4).

More recently, Wang and colleagues [126], using an ex vivo approach by plasma/serum samples collected from healthy subjects administered with goldenseal extracts, have confirmed the inhibitory effects towards CYP3A-catalyzed midazolam 1′-hydroxylation. Particularly, the authors have calculated an IC_50_ value of 33.03 μM for hydrastine and 473.0 μM for berberine, indicating that hydrastine is more potent in terms of inhibiting CYP3A [126]. Moreover, concurrent, albeit moderate, inhibition of P-gp in humans [127] might contribute increasing drug exposure in subjects assuming also goldenseal.

#### 2.2.2. Kava Kava

Kava kava (*Piper methysticum*) extract has been shown to inhibit several CYPs in vitro, including 3A4, 2C9, 2C19, 1A2, 2D6, but not 2E1, 2A6 and 2C8 [128]. On the other hand, Zou and co-workers (2004) [129] have shown inhibition of CYP2E1, too. Nevertheless, in healthy volunteers, the treatment with kava extracts for 28 days (at 138 mg/day kava lactones) or 14 days (at 253.5 mg/day kava lactones) did not influence CYP2D6, CYP1A2, or CYP3A4/5 activity as determined by single-time point phenotypic metabolic ratios [124,130]. Likewise, kava supplementation (253.5 mg/day kava lactones for 14 days) did not induce significant effects in a clinical study on the pharmacokinetics of CYP3A4 substrate drug, midazolam [125].

Treatment with kava extract for 28 days at a dose of 138 mg/day kava lactones induced a statistically significant reduction (about 40%) in CYP2E1 activity [124], confirming in vitro results of Zou et al. [129] but contrasting those of Mathews et al. [128]. Finally, the available in vivo data suggest that more studies are necessary to clearly consider or exclude the capacity of kava to inhibit drug metabolism in humans (Table 4). Meanwhile, the co-administration of kava with drugs undergoing CYP-mediated metabolism should be closely supervised to prevent possible inhibition of their biotransformation which may pose the patients at higher risk of adverse effects or toxicity of drug.

#### 2.2.3. Echinacea

The ability of *Echinacea* extracts to inhibit the activity of human cytochrome P450 enzymes is unequivocally reported in several studies in vitro [120,131,132,133,134,135]. However, different variables including the species (*E. angustifolia, E. purpurea, E. pallida*), plant parts (aerial parts, whole plants, roots or a combination of them), and the kind of preparations (such as infusion, tincture, tablets, capsules, methanolic extract or liquid capsules) do not allow comparison of the various studies. Accordingly, when considering CYP inhibition by 10 different commercially available *Echinacea* preparations, it was shown that all tested extracts were capable to inhibit CYP3A4, but inhibitory potencies (expressed as median inhibitory concentration, IC_50_) changed by a factor of 150 [133]. Then, a lack of information or insufficient characterization regarding the investigated extract, definitely preclude any translation of in vitro results to clinical setting. Actually, different studies examined in humans the effects of various *Echinacea* extracts on cytochrome P450 activity [130,136,137,138,139,140]. In healthy volunteers, the intake of *E. purpurea* extract from root (400 mg, four times daily for 8 days) significantly reduced the oral clearance (Dose/AUC _(0-∞)_) of the CYP1A2 probe caffeine by 27%; this effect occurred with a significant reduction by 11% of the 6-h serum paraxanthine-to-caffeine concentration ratio along with an increase in C_max_ (by 30%) and T_max_ from 3 to 4 h [136]. A similar, but not statistically significant result was reported by Gurley et al. (2004) [137] that observed in human volunteers a reduction (by 13%) of the 6-h serum paraxanthine-to-caffeine concentration ratio treated for 28 days with an *Echinacea purpurea* whole plant extract (800 mg two times daily). Altogether, this evidence indicates an inhibitory effect of *Echinacea* on CYP1A2 activity in vivo. Nevertheless, the result reported by Gurley et al. [137] that changes in 6-h serum paraxanthine-to-caffeine concentration ratio did not reach statistical significance, and the high degree in inter-individual variability documented by Gorski et al. [136], induce to consider with caution the clinical relevance of this evidence.

Although additional studies are definitely necessary before an assertion can be done about the ability of *Echinacea* to inhibit the metabolism of CYP1A2 substrate drugs in humans, it is recommended to take with caution *Echinacea* extracts with such drugs. Similar conclusions can be made when *Echinacea* supplementation is assumed with CYP2C9 substrate drugs following the observation that the administration of *Echinacea purpurea* extract (400 mg four times a day for 8 days) increased the AUC of oral tolbutamide and reduced its oral clearance [136]. However, intake for 2 weeks (four times daily) of MediHerb Premium Echinacea TM tablets (a mixture of *Echinacea angustifolia* and *Echinacea purpurea*) weakly increased the apparent clearance of (S)-warfarin [138]. No significant modifications were observed in CYP2D6 activity in three different studies after the administration of *Echinacea* extracts [130,136,137]. Likewise, no significant changes in CYP2E1 activity were observed by Gurley et al. [137] following the oral administration of *Echinacea*. Instead, confusing results have been shown on the effects of *Echinacea* administration on the disposition of drugs substrate of CYP3A4 isoform. *Echinacea purpurea* whole plant extract (800 mg, two times daily) for 28 days did not induce changes in pre- and post-supplementation values of l-hydroxymidazolam/midazolam serum ratio 1 h after oral administration of midazolam (8 mg) to 12 healthy volunteers [137]. Similar results were observed by Gorski and collaborators [136] on pharmacokinetic (AUC, oral clearance, C_max_, T_max_, t_½_) values of midazolam (5 mg) in 12 healthy subjects before and after treatment with *Echinacea purpurea* root extract (400 mg, four times daily for 8 days). However, these same authors reported that *Echinacea* administration significantly influenced pharmacokinetics of midazolam, administered intravenously, causing a decrease in AUC_(0-∞)_ by 23% and in t_½_ by 58% and an increase in systemic clearance by 42% that could suggest an inducing activity on hepatic CYP3A4 [136]. Furthermore, it was calculated a significant increase by 43% of oral bioavailability of midazolam as determined by the ratio of the dose-normalized AUCs after intravenous and oral drug administration [136]. These results would suggest that drug oral availability is increased by the inhibition of intestinal CYP3A4 but this conclusion is not supported by the evidence that no changes in oral AUC values were observed between pre- and post-supplementation with *Echinacea*. Incidentally, in thirteen healthy volunteers an increase was measured in the apparent oral clearance of midazolam by 37% and a reduction of t_½_ by 45% after treatment with *Echinacea purpurea* (Echinamide^®^ Natural Factors capsule) (250 mg twice daily for four weeks) reflecting no effects of *Echinacea* on both intestinal and hepatic CYP3A enzyme activity [139]. Longer *Echinacea* treatment (four weeks versus 8 days) used by Penzak and colleagues [139], the composition of extracts used and differences in CYP3A phenotyping methods could explain the induction or no effect on enzyme activity observed by Penzak et al. [139] and Gorski et al. [136], respectively.

More recently, Goey and colleagues [140] reported no changes in docetaxel AUC_(0–∞)_, *t*_1/2_ and C_max_ pre- and post-infusion values in cancer patients after treatment with Echinaforce^®^ three times daily for fourteen days [140]. Similar results were obtained in HIV-infected patients where *Echinacea* treatment, every 6 h for 28 days did not show an effect on C_max_ and AUC _(0-24)_ of etravirine (ClinicalTrials.gov:NCT01347658) [141], darunavir and ritonavir (ClinicalTrials.gov:NCT01046890) [142]. The limited number of patients included in the studies requires such results to be taken with great caution also in light of the fact that, after Echinacea treatment, individual patients did show a decrease in darunavir concentration at the end of the dosing interval (40%) and area under the time-concentration curve (30%) [142]. Moreover, additional experiments are clearly necessary to clarify whether or not *Echinacea* may inhibit intestinal CYP3A4 and induce hepatic CYP3A4 along with the identification of the active compounds and the underlying mechanisms. An in vitro study showing the effects of an *Echinacea* preparation and isolated alkylamides on CYP3A4 expression did not document any statistically significant changes in the mRNA steady-state level of CYP3A4 after treating human hepatic cellular carcinoma HepG2 cells with clinically relevant concentrations of Echinaforce^®^ or any of the alkylamides [133]. Conversely, in the same cell line, positive results were obtained more recently that suggest gene expression up-regulation of metabolic enzyme and transporter by activation of PXR pathway [143,144]. In co-transfected HepG2 cells using the luciferase gene reporter assays, it was observed that *Echinacea* extract induced a time- and concentration-dependent activation of CYP3A4, CYP1A2 enzymes and the ABCB1 drug transporter [143]. Successively, the same authors showed that *Echinacea* exerts a regulatory effect on the microRNA expression profile of the human drug transporter ABCG2 in HepG2 cells and that miR-655-3p acts as a posttranscriptional regulator [144]. It is known that two different nuclear receptors are involved in the regulation of ABCG2 and ABCB1; however, there is some evidence that Ahr, which regulates ABCG2 expression, has similarities with PXR, including intracellular localization, ligand binding or activation, nuclear localization and activation of target genes. Thus, a possible PXR/Ahr cross-talk mechanism of the *Echinacea-purpurea*-mediated activation of ABCB1 and ABCG2 genes was speculated [144], but further studies are necessary to confirm this hypothesis. Although in vitro studies reported confusing results, which can be disclosed from studies in healthy volunteers is that changes in CYP3A4 substrate drug disposition may occur possibly due to concomitant inhibition of intestinal and induction of hepatic metabolism or transporters depending on duration of *Echinacea* treatment. More complex and unforeseeable interactions may be pictured depending on the relative burden of intestinal and hepatic metabolism in predicting the overall availability of a specific drug. However, in the light of these results, it sounds strange the lack of case reports in the medical literature implicating *Echinacea* supplementation in altering the pharmacologic response of prescribed drugs, although the wide use of this herbal medicine.

#### 2.2.4. Milk Thistle

Flavolignans from milk thistle (*Silybum marianum*) extract are reported as inhibitors of CYP3A4, CYP2C9 GT1A1, GT1A8, GT1A10 enzymes [145,146,147,148] and/or P-gp [149,150]. The main component of the *Milk thistle* extract, obtained from the seeds of the plant, is silymarin, which is a mixture of many flavonolignans, including silybin, silycristrin and silydianin. Silybin, the main constituent of silymarin, has been reported to inactivate purified, recombinant CYP2C9 and CYP3A4 enzymes. Specifically, a metabolism-dependent inactivation lead to the generation of reactive silybin intermediate that alkylated the heme moiety of the enzymes ([145] and references therein). Besides, different results were achieved when silymarin was administered with drugs that behave as substrates of CYP3A, CYP2C9 and/or P-gp in controlled clinical studies in healthy subjects (Table 4). The oral intake of silymarin (153 mg three times/day for 3 weeks) did not produce a significant effect on the pharmacokinetics of the CYP3A4/P-gp substrate indinavir except a reduction by 25% of the trough hour-8 level [151]. Successively, Di Cenzo and co-workers (2003) [152] observed a rather similar reduction of the trough plasma concentration of indinavir in healthy subjects after treatment with silymarin at the dose of 160 mg/day for 2 weeks. Interestingly, Rajinarayana and colleagues [153] indicated that the administration of silymarin (140 mg/day for 9 days) in healthy subjects decreased the AUC_(0-48)_, C_max_, and the t_½_ of metronidazole, a drug metabolized by cytochrome P450 isozymes, a result suggestive of an inducing effect of herbal supplementation on intestinal and hepatic enzymes. However, silymarin failed to increase CYP3A4 mRNA levels in human hepatocytes or primary cultures [154]. Therefore, intake of milk thistle (175 mg, twice daily, standardized to 80% silymarin) for 28 days did not influence the pharmacokinetics of the CYP3A4 substrate midazolam as determined by 1-hydroxymidazolam/midazolam serum ratios (1-h sample) [137]. This result was confirmed by the same authors [155] by using canonical multiple post-dose serum concentrations analysis. In fact, administration for 14 days of milk thistle (300 mg, three times daily, standardized to contain 80% silymarin) did no induce changes in the AUC_(0-∞)_, C_max_ and T_max_ CL/F, t_½_ after oral administration of midazolam [155].

Intriguingly, van Erp and co-authors (2005) [156] investigated the effects of milk thistle extract (200 mg containing 80% silymarin, thrice daily) in six cancer patients on the pharmacokinetics of irinotecan, substrate for CYP3A4 and UGT1A1. The results indicated that intake of milk thistle did not reach a significant effect on irinotecan clearance though the relative extent of conversion of irinotecan to its active CYP3A4-derived metabolite SN-38 (i.e., AUC_SN38_/AUC_irinotecan_) was slightly, but significantly, reduced by about 13% and 16% after short-term (4 days) or more prolonged (12 days) administration of the extract, respectively. On the other hand, silymarin did not influence the relative extent of glucuronidation of SN-38 (i.e., AUC_SN-38G_/AUC_irinotecan_) [156]. However, in fifteen HIV-infected patients, intake of silymarin (150 mg every 8 h) for 14 days induced a slight decrease in the AUC_(0-12)_ and C_max_ of darunavir and ritonavir (ClinicalTrials.gov:NCT01346982) [157]. Furthermore, no statistically significant differences were observed in healthy volunteers for AUC_(0-12)_ and C_max_ values of midazolam, caffeine, tolbutamide and dextromethorphan calculated pre and post oral intake of silymarin (140 mg) three times daily for 14 days [158]. These drugs were used as probes to evaluate the effects of the extract on the activity of CYP3A4/5, CYP1A2, CYP2C9, CYP2D6, respectively [158]. Furthermore, the authors measured plasma levels of silybin B with a C_max_ of 0.049 μM. Intriguingly, this is a very low value compared to the IC_50_ of 60 µM, necessary to inhibit CYP-3A4 isoform and calculated by Brantley and colleagues [159] for the favolignan in human liver and intestinal microsomes. The necessity of high plasma levels of the extract natural constituents to inhibit drug metabolism was confirmed by Wang et al. [160]. In fact, in a preclinical study in rat a dose of 50 mg·kg^−1^ of silybin for 15 consecutive days increases AUC_(0−t)_, AUC_(0−∞)_ and t_1/2_ of imatinib [160]. Han and co-workers [161] reported in humans an engaging evidence of silymarin-mediated inhibition of CYP2C9 in a study regarding the pharmacokinetics of losartan and E-3174, CYP2C9-derived active drug metabolite, in subjects with different CYP2C9 genotypes before and after the administration of silymarin (420 mg/day for 14 days). Silymarin induced a significant increase in the AUC_(0-24)_ (by 109%), AUC_(0-∞)_ (by 108%) and C_max_ (by 89%) of losartan and a significant decrease (by 51%) in the oral clearance of the drug (estimated as the ratio CL [dose/AUC_(0-∞)_] in patients genotyped as CYP2C9*1/*1 wild-type homozygotes), but not in those with the CYP2C9*1/*3 genotype (the CYP2C9*3 allele being associated with lower enzyme activity). Regarding the metabolic product, changes in these parameters were correlated with a significant reduction in the C_max_, AUC_(0-24)_ and AUC_(0-∞)_ of E-3174. The potential of milk thistle to influence the pharmacokinetics of P-gp substrate drugs has been reported in few studies. No significant effect was reported by Gurley and co-workers [162] after intake of milk thistle (300 mg, three times daily, standardized to contain 80% silymarin) for 14 days on the pharmacokinetics of digoxin. On the contrary, it has been subsequently reported that in healthy Chinese subjects, silymarin (140 mg three times daily for 14 days) significantly enhanced the AUC_(0-36)_, the AUC_(0-∞)_ and the C_max_ and significantly decreased the oral clearance (CL/F) of talinolol, a P-gp substrate drug, but did not influence the t_½_ [163]. The gathered evidence suggests that more investigations are required to clarify the capacity of silymarin to modify the pharmacokinetics of co-administered drugs. It is important to highlight the potential of drug interaction after supplementation with the high doses of silibinin (13 g of oral silybin-phytosome daily), which are advised in prostate cancer patients for phase II study as these give rise to plasma silibinin levels higher than those previously reported at standard dosage. Accordingly, a pharmacokinetic and phase I study was performed with high doses of a commercially available silibinin-phosphatidylcholin complex, to measure a phase II dose recommendation and investigate the toxicity of high-dose therapy in prostate patients [164]. The results indicated that some patients have peak plasma levels of silibinin in excess of 100 µM and reported bilirubin elevation consistent with UGT-1A1 inhibition [164].

## 3. Discussion

Medicinal plant extracts, useful for different therapeutic purposes, e.g., aromatherapy in dementia [165,166,167,168], may influence the pharmacokinetics of co-administered drugs then inducing modifications in plasma drug levels. Consequently, it may not reach a therapeutic response or, alternatively, it may cause drug-induced toxicity. Interaction happens usually during all the pharmacokinetic phases of a drug (intestinal absorption, distribution, hepatic elimination and/or renal excretion). Several clinical studies report that herbal extracts may alter oral availability and/or the systemic hepatic clearance of drugs co-administered but the effects on drug distribution and renal excretion are not yet largely investigated. Different mechanisms are involved in herb–drug interactions as membrane transport, drug metabolizing enzymes or both. However, while many clinical studies carried out have investigated the potential for P-gp- and/or CYP-mediated interaction, studies regarding interaction involving a drug transporter other than P-gp and phase II metabolism are missing. The attempt made to amplify knowledge about the induction or inhibition of transporters or metabolic enzymes by natural extract compounds allows us to anticipate potential drug–herbal interactions and reduces the risk of therapeutic failure or drug toxicity. In this paper, two major limitations that influence ability to anticipate interactions between herbal medicines and drugs are reviewed and discussed. One aspect refers to the effort in translating data from in vitro studies to clinical environment. For example, SJW extracts and their components, hypericin, hyperforin, 13,II8-biapigenin and quercetin, have been recorded to be competitive or non-competitive inhibitors of several CYP enzymes in vitro [169]. Indeed, SJW is the herbal medicine for which extensive literature indicates its ability to induce P-gp and CYP3A4 in humans. Indeed, SJW poses the risk of interaction with indinavir or other drugs metabolized by cytochrome P450. The example provided by SJW demonstrates that only one herbal medicine can induce interactions through several mechanisms. Moreover, *Silybum marianum* influences other drugs both at the level of cytochrome P450 and of P-gp. Another aspect refers to the contrasting results often shown in clinical studies. In fact, discrepancy between in vitro and clinical results may occur and this can be explained by several reasons: (1) the use of high concentrations of herbal product in vitro not achievable in humans after the administration of the conventional dose; (2) the modification of enzyme activity in the in vitro setting (ionic strength, pH changes, use of solvents, etc.) respect to clinical administration; (3) the lack of oral bioavailability, protein-binding properties or in vivo formation of metabolites during in vitro studies. For all the listed reasons, well-designed clinical trials, a suitable choice of substrate drugs for transporters and phase I and phase II enzymes, the standardization of herbal extracts [170], along with the genotyping of drug-metabolizing enzymes that display clinically important polymorphisms, should aid to overcome in the near future some inconsistent results originating from clinical studies.

Further clinical studies are needed in the near future to ensure safety on the use of medicinal plants in combination with conventional medicine. Moreover, it is important to emphasize that it is absolutely necessary to comply with existing guidelines on how to conduct clinical trials on the interactions between natural extracts and synthetic drugs. More studies are also needed to elucidate all possible mechanisms of herbal medicine–drugs interactions, most of the actual data being focused on interactions involving metabolic enzymes and carriers.

## 4. Methods

The literature search has been conducted in PubMed, Cochrane and ClinicalTrials.gov up to March 2020, matching the name of the herbal extract of interest with the key words: “pharmacokinetic parameters” and/or “clinical studies” and “drug interactions”. The herbal extracts included in the search are the most widely used [1] and long-studied for drug interactions: *St John’s wort, Ginkgo biloba, Allium sativum, Hydrastis Canadensis, Kava kava, Echinacea* and *Milk thistle*. The results have been filtered in order to find literature not included in the previous search. All authors checked the abstracts for interaction studies independently and reviewed the findings. The included results are controlled pharmacokinetic clinical studies recruiting patients or healthy participants and with sequential treatment, randomized or crossover study design. However, in vitro and in vivo studies supporting or speculating mechanism of action of the constituents in the herbal extracts have also been included. The study endpoints considered relevant are: clearance, apparent clearance or area under the concentration–time curve (AUC) after an appropriate period of pre-treatment with the herbal extracts. Eligible articles have been evaluated to highlight when a significant interaction was reported, including the mean extent of change in the systemic exposure to assess clinically meaningful interactions.

## Figures and Tables

**Table 1:** Examples of clinical evidence of St John’s wort (SJW)–drug interaction mediated mainly by induction in healthy volunteers or patients *.

Hypericum Extract		Subject Number	Drug Pharmacokinetic Parameters	Protein Involved	Ref.
*Dose* 3 × 300 mg/day	*Duration*				
LI160 Rex Sund Jarsin LI160 Jarsin LI160 Jarsin LI160 Jarsin LI160	10 days 12 days	13 male and 12 female 12 female 13 subjects 16 male and 4 female 10 male and 11 female 9 male	↓ AUC and *C*_max_ of digoxin ↓ AUC and *C*_max_ of midazolam ↓ AUC of bosentan ↓ AUC and *C*_max_ of ambrisentan ↓ *C*_max_ of midazolam and fexofenadine ↓ AUC and *C*_max_ of talinolol	P-gp CYP3A4 CYP2C9/3A4 CYP3A4/5 CYP2C19 CYP3A4 and P-gpCYP3A4, P-gp	[15] [56] [57] [43] [51]
Buyers Jarsin LI160 TruNatur LI160 Kira Buyers Buyers Solaray 3 × 325 mg/day Movina Jarsin LI160 Kira Kira Willmar Schwabe Pharm Jarsin LI160 Hyperiplant	14 days	6 male and 2 female 7 male and 5 female 8 male 12 subjects * 6 male and 6 female 12 male 12 male 15 male 8 male 16 male 14 male 15 male and 6 female 14 subjects 6 male and 6 female 4 male and 7 female *	↓ AUC of indinavir ↓ AUC of midazolam ↓ AUC of simvastatin ↓ AUC of amitriptyline ↓ AUC and *C*_max_ of imatinib ↓ AUC and *C*_max_ of omeprazole ↓ AUC and *C*_max_ of mephenitoin AUC and *C*_max_ no change of repaglinide ↓ AUC and *C*_max_ of verapamil ↓ AUC and *C*_max_ of voriconazole ↓ AUC and *C*_max_ of zolpidem ↓ AUC and *C*_max_ of gliclazide ↓ AUC and *C*_max_ of nifedipine ↓ AUC and *C*_max_ of S-ketamine ↓ AUC of docetaxel	CYP3A4 CYP3A4 CYP2C8/3A4 CYP3A4 CYP3A5, P-gp CYP2C19 CYP2C19 CYP2C8/3A4 CYP3A4 CYP3A4/5, P-gp CYP2C19 CYP3A4 CYP-2C9 CYP3A4 CYP3A4/2B6 CYP3A	[13] [33] [36] [37] [45] [46] [47] [66] [49] [50] [55] [52] [53] [54] [58]
LichtwerPharma AG	18 days	2 male and 8 female *	↓ AUC and *C*_max_ of tacrolimus	CYP3A, P-gp	[48]
Modigen 2 × 300 mg/day	21 days	20 male	no effect steady-state of metformin		[62]
Rexall Sundown Buyers -	56 days 28 days	12 female 16 female 6 male and 6 female	↓ AUC and *C*_max_ of ethinyl estradiol/norethindrone combination ↓ AUC of ethinyl estradiol/norethindrone combination ↓6-hydroxychlorzoxazone/chlorzoxazone serum ratios (2-h) ↓1-dydroxymidazolam/midazolam serum ratios (1-h) in *young* and in *elderly* subjects	CYP3A CYP3A4 CYP2E1 CYP3A4	[41] [42] [38] [39]
-	31 days	2 male and 2 female *	↓of (R,S)-methadone concentration-to-dose ratios		[40]

**Table 2:** Clinical pharmacokinetic studies of Gingko biloba–drug interaction mediated mainly by induction in healthy volunteers or patients *.

Herbal Extract		Subject Number	Drug Pharmacokinetic Parameters	Protein Involved	Ref.
*Dose*	*Duration*				
**Ginkgo biloba** extract 240 mg standardized to 0.12% to 0.3% hypericin	14 days	12 subjects	↓ AUC of alprazolam	CYP3A4	[77]
			no effect		
			half-life of elimination		
Ginkgo biloba extract 240 mg	28 days	14 subjects	↓ AUC and *C*_max_ of midazolam	CYP3A4	[78]
EGb 761 360 mg standardized to the content of 24% Ginkgo flavone glycoside and 6% terpene lactone	28 days	10 male	↓ oral clearance ↑AUC of midazolam ↓ AUC of tolbutamide	CYP3A4 *Inhibition* CYP2C9	[80]
Ginkgo biloba extract mg not available	some months	1 HIV-infected *	↓ plasma concentrations of efavirenz	CYP2B6	[87]
Ginkgo biloba extract 120 mg, twice daily	12 days	7 male	voriconazole no pharmacokinetic change of	extensive (2C19*1/2C19*1) and poor (2C19*2/2C19*2) metabolizers	[85]
Ginkgo biloba extract 120 mg, twice daily	14 days	14 male	bupropion no pharmacokinetic change	CYP2B6	[88]
Ginkgo biloba extract 120 mg, twice daily	28 days	12 male	diazepam no pharmacokinetic change	CYP2C19	[86]
Ginko biloba extract 360 mg/day	14 days	10 male	↑*C*_max_ and AUC of talinolol no effects elimination half-life	P-gp *inhibition*	[90]
Tavonin extract 120 mg twice daily	14 days	9 male and 9 female	↑*C*_max_ and AUC of raltegravir	P-gp *inhibition*	[92]
EGb 761^®^ 120 mg twice daily or 240 mg once daily	8 days	18: male and female	caffeine tolbutamide omeprazole dextromethorphan midazolam no pharmacokinetic changes	CYP1A2 CYP2C9 CYP2C19 CYP2D6 CYP3A	[95]
Ginexin^®^ 80 mg twice daily	7 days	34 male	Cilostazol no pharmacokinetic changes	CYP2C19 and CYP3A	[96]
Ginkgo biloba extract 360 mg)	14 days	16 male	↓AUC_(0–∞)_ and *C*_max_ ↑ Vd and CL atorvastatin	CYP3A4	[97]
Ginko biloba extract 120 mg twice daily	14 days	14 subjects	↑AUC_(0–∞)_ and *C*_max_ simvastatin	OATP1B1, CYP3A4 and BCRP	[99]

**Table 3:** Clinical pharmacokinetic studies of interaction between garlic extracts and drugs mediated mainly by induction in healthy volunteers or patients *.

Herbal Extract		Subject Number	Drug Pharmacokinetic Parameters	Protein Involved	Ref.
*Dose*	*Duration*				
Garlic oil 500 mg three times daily	28 days	6 male and 6 female	↓6-hydroxychlorzoxazone/chlorzoxazone serum ratios (2-h) in young and in elderly subjects ↓paraxanthine/caffeine serum ratios (6-h) 1-dydroxymidazolam/midazolam serum ratios (1-h) debrisoquine urinary recovery ratios *no changes*	CYP2El CYP1A2 CYP3A4 CY2D6	[38] [39]
Garlic powder tablets (kwai) 3 × 600 mg tablets twice daily	14 days	9 male and 6 female	AUC, *C*_max_, T_max_, half-life of elimination of alprazolam urinary dextromethorphan/dextrorphan ratios activity *no changes*	CYP34 CYP2D6	[113]
Garlic powder caplets twice daily	19 days	4 male and 6 female	↓*C*_max_ and AUC of saquinavir	P-gp	[114]
Garlic capsules twice daily	4 days	5 male and 5 female	not pharmacokinetics changes of ritonavir		[116]

**Table 4:** Clinical pharmacokinetic studies of herbal–drug interactions mediated mainly by inhibition in healthy volunteers, cancer* or HIV patients.

Herbal Extract		Subject Number	Drug Pharmacokinetic Parameters	Protein Involved	Ref.
*Dose*	*Duration of treatment*				
**Hydrastis Canadensis** root extract [Goldenseal] 900 mg, 3 times daily (no standardization claim)	28 days	6 male and 6 female	debrisoquin and midazolam pre- and post-supplementation phenotypic ratio means	CYP2D6 and CYP3A4/5	[124]
Goldenseal 1.323 mg, 3 times daily (standardized to contain 24.1 mg isoquinoline alkaloids per capsule)	14 days	8 male and 8 female	↑ *C*_max_, AUC_(0-∞,)_ elimination half-life of midazolam	CYP3A4/5	[125]
**Kava-kava** extract138 mg/die	28 days	6 male and 6 female	chlorzoxazone pre- and post-supplementation phenotypic ratio means		[124]
**Echinacea purpurea** extract from root 400 mg, 4 times dail	8 days	6 male and 6 female	↓ Dose/AUC (0-∞) ↑ *C*_max_ and T_max_ of caffeine	CYPlA2	[136]
Echinacea purpurea whole plant extract 800 mg 2 times daily	28 days	6 male and 6 female	6-h serum paraxanthine-to-caffeine concentration ratio	CYP1A2	[137]
Echinacea purpurea extract from root 400 mg, 4 times daily	8 days	6 male and 6 female	↑ AUC of tolbutamide	CYP2C9	[136]
MediHerb Premium Echinacea^TM^ tablets 4 times daily (mixture of Echinacea angustifolia and Echinacea purpurea)	14 days	12 male	↑apparent clearance of (S)-warfarin	CYP2C9	[138]
Echinacea purpurea extract from root 400 mg, 4 times daily	8 days	6 male and 6 female	↓ AUC(0-∞) by 23% and of t_½_ of midazolam, administered intravenously	CYP3A4	[136]
Echinamide^®^ Natural Factors capsule 250 mg 2 daily	28 days	8 male and 5 female	↑ AUC of midazolam by 37% and a reduction of half-life by 45%	CYP3A4	[139]
Echinaforce^®^ 3 times daily	14 days	9 male and 2 female*	AUC_(0–∞)_, t_1/2_ and *C*_max_ of docetaxel No effect	CYP3A4	[140]
Arkocapsulas Echinacea capsule 500 mg every 6 h	14 days	14 male and 1 female°	AUC and *C*_max_ of darunavir and ritonavir No effect	CYP3A4	[142]
Arkocapsulas Echinacea capsule 500 mg every 6 h	14 days	14 male°	AUC and *C*_max_ of etravirine No effect	CYP3A4	[141]
**Silybum marianum (milk thistle)**					
Silymarin (Thisilyn) 153 mg 3 times daily	21 days	12 subjects	! *C*_8_(μg/mL) of indinavir	CYP3A4	[151]
Silymarin 160 mg	14 days	7 male and 3 female	! *C*_max_, AUC of indinavir	CYP3A4	[152]
Milk thistle 175 mg 2 daily (standardized to 80% silymarin)	28 days	6 male and 6 female	1-hydroxymidazolam/midazolam serum ratios (1-h sample) No effect	CYP3A4	[137]
Milk thistle 300 mg 3 times daily (standardized to contain 80% silymarin)	14 days	10 male and 10 female	AUC_(0-∞)_, *C*_max_ and *T*_max_ CL/F, t_½_ of midazolam No effect	CYP3A4	[155]
Milk thistle extract 200 mg 3 times daily (standardized to 80% silymarin)	4 or 12 days	2 male and 4 female *	No effects on irinotecan clearance	CYP3A4 UGT1Al	[156]
Silymarin (Legalon) 150 mg every 8 h	14 days	15 patients°	AUC and *C*_max_ of darunavir and ritonavir No effect	CYP3A4	[157]
Silymarin (Legalon) 140 mg 3 times daily	14 days	12 subjects	AUC_(0-12)_ and *C*_max_ of midazolam, caffeine, tolbutamide, dextromethorphan No effect	CYP3A4/5, CYP1A2, CYP2C9, CYP2D6	[158]
Silymarin 420 mg/day	14 days	12 male	↑AUC_(0-24)_, AUC_(0-∞)_ and *C*_max_ dose/AUC_(0-∞)_ of losartan	CYP2C9*1/*1	[161]
Silymarin (140 mg three times daily)	14 days	18 male	↑AUC_(0-36)_, the AUC_(0-∞)_ and *C*_max_ ↓oral clearance (CL/F) of talinolol	Pg-p	[163]
